# Centralised and Decentralised Sensor Fusion-Based Emergency Brake Assist

**DOI:** 10.3390/s21165422

**Published:** 2021-08-11

**Authors:** Ankur Deo, Vasile Palade, Md. Nazmul Huda

**Affiliations:** 1 Department of Autonomous Driving, KPIT Technologies, Pune 411057, India; ankur.deo@kpit.com; 2 Centre for Data Science, Coventry University, Priory Road, Coventry CV1 5FB, UK; 3 Department of Electronic and Electrical Engineering, Brunel University London, Kingston Ln, Uxbridge UB8 3PH, UK; nazmul.huda@brunel.ac.uk

**Keywords:** sensor fusion, autonomous driving, ADAS, object detection and tracking

## Abstract

Many advanced driver assistance systems (ADAS) are currently trying to utilise multi-sensor architectures, where the driver assistance algorithm receives data from a multitude of sensors. As mono-sensor systems cannot provide reliable and consistent readings under all circumstances because of errors and other limitations, fusing data from multiple sensors ensures that the environmental parameters are perceived correctly and reliably for most scenarios, thereby substantially improving the reliability of the multi-sensor-based automotive systems. This paper first highlights the significance of efficiently fusing data from multiple sensors in ADAS features. An emergency brake assist (EBA) system is showcased using multiple sensors, namely, a light detection and ranging (LiDAR) sensor and camera. The architectures of the proposed ‘centralised’ and ‘decentralised’ sensor fusion approaches for EBA are discussed along with their constituents, i.e., the detection algorithms, the fusion algorithm, and the tracking algorithm. The centralised and decentralised architectures are built and analytically compared, and the performance of these two fusion architectures for EBA are evaluated in terms of speed of execution, accuracy, and computational cost. While both fusion methods are seen to drive the EBA application at an acceptable frame rate (~20 fps or higher) on an Intel i5-based Ubuntu system, it was concluded through the experiments and analytical comparisons that the decentralised fusion-driven EBA leads to higher accuracy; however, it has the downside of a higher computational cost. The centralised fusion-driven EBA yields comparatively less accurate results, but with the benefits of a higher frame rate and lesser computational cost.

## 1. Introduction

In today’s state-of-the-art technology, the application of multiple sensors that are fine tuned to perceive the environment precisely is seen as instrumental for increasing road safety [1,2]. Thanks to robust and reliable exteroceptive sensors, such as the LiDAR sensor [3], the radio detection and ranging (RADAR) sensor [4], cameras, and ultrasonic sensors [5], amongst several others, intelligent vehicles are capable of accurately perceiving the environment around them [2]. This allows them to anticipate and/or detect emerging dangerous situations and threats.

In case of mono-sensor applications, the system is prone to errors, as failure of the only available sensor can lead to breakdown of the entire system [6]. Having multiple sensors with different field-of-views and capabilities often helps in making the system more robust, as the system can still operate with acceptable efficacy after failure of one or more sensors from an agglomeration of multiple sensors [6,7]. Different sensors have different levels of reliability under a multitude of scenarios. For example, the performance of a camera sensor is deteriorated substantially in dark conditions. Thus, the probability of false positives or false negatives increases under such circumstances [8,9]. A LiDAR sensor is relatively robust under dark situations, thereby allowing for more dependable detections. However, it has certain drawbacks as it cannot recognise the colour of the detected object [10]. Hence, applications like ‘traffic sign recognition’ cannot be carried out by using LiDAR alone. In such cases, use of a camera sensor is mandatory [8,11].

Multi-sensor fusion is the process of combining data from multiple sensors so that the cumulative data are enhanced in terms of reliability, consistency, and quality, compared to the data that would be acquired from a single sensor [12,13]. In this paper, we focus on ‘object-level’ or ‘high-level’ multi-sensor fusion techniques for emergency brake assist (EBA) systems. First, we concentrate on the importance of a multi-sensor fusion approach, followed by an exegesis of ‘centralised’ and ‘decentralised’ object-level multi-sensor fusion to drive the EBA feature. Once the environment is fully perceived, with the present conditions known and future conditions estimated, the vehicle software can then undertake proactive decisions and actions to either avoid the upcoming threat, or, in case the situation is inevitable, boost safety of the driver and other occupants [12,13]. Thus, ADAS applications utilising multi-sensor fusion as their backbone have potential to make mobility safer and more efficient [14,15].

Emergency brake assist (EBA) is an ADAS system that assists the driver in avoiding a collision or decreasing the impact of collision with other vehicles or vulnerable road users when the collision is unavoidable [16,17]. Research shows that in many critical situations, human drivers tend to react either too late or in a wrong way [18]. In such scenarios, the best alternative is to apply the vehicle brakes with the safe maximum force to minimise the consequences of the unavoidable impact [19]. EBA primarily consists of two parts [20]:

(1) Detection: identify critical scenarios which can lead to an accident and warn the driver accordingly through audio and/or visual indications;
(2) Action: in scenarios where impacts or accidents are inevitable, EBA can decrease the speed of the ego-vehicle by applying brakes in advance to achieve minimal impact.

In this paper, we will only focus on the ‘detection’ part of EBA. For EBA to function appropriately, a precise environment perception is required. As a result, a reliable and consistent sensor fusion network is necessary to drive this algorithm [21]. For this paper, the EBA is designed such that an alert shall be displayed on the dashboard notifying that either a critical or a safe scenario has been detected.

The work outlined in this paper contributes to the research by:

1. Highlighting the advantages and challenges of using multi-sensor fusion driven ADAS algorithms over mono-sensor ADAS features.
2. Providing analytical comparisons of the two proposed methodologies of sensor fusion—‘centralised’ and ‘decentralised’ fusion architectures.
3. Implementing an EBA algorithm and critically analysing the behaviour, performance, and efficacy of the feature driven by the two proposed fusion methods.

The paper is structured as follows: Section 1 provides the introduction and motivation behind this work. Section 2 presents the literature review, where a multitude of papers and work done by various researchers is critically studied and analytically compared. Section 3 sheds light on the proposed methods for sensor fusion alongside the sensor building blocks, such as the camera and LiDAR object detection, tracking and fusion algorithms, types and methods of sensor fusion, and their fundamentals. This part covers the theory required to implement the multi-sensor fusion-based EBA feature. Section 4 shows the implementation of EBA and analytically compares the performance of centralised and decentralised fusion-driven EBA, based on execution speed, accuracy, noise immunity, and computational cost. Section 5 concludes the work.

## 2. Background and Previous Work

### 2.1. Related Work

Sensor fusion targets a variety of applications in the automotive domain. The architecture of the fusion algorithm and the methodology, and the amount and type of sensors used depend on the task to be performed and the sensitivity and criticality of the parent system. Sensors such as cameras, LiDAR, ultrasonic sensors, and RADAR can be used to perceive the environment around the ego-vehicle under different circumstances. An efficient technology, which involves fusing information from a point cloud (generated by LiDAR) and an image (generated by camera), is discussed by Kocic et al. [18]. Accordingly, we shall use the LiDAR–camera combination of sensors in our work. By using a similar fusion architecture, the localisation and mapping can also be done; however, we shall restrict the scope of our work to the construction of an alert-based EBA system only.

The work done by Herpel et al. [1] presents a systematic and detailed analysis of high-level (object-level) and low-level sensor fusion. Low-level sensor fusion techniques usually involve heavy computation and are more susceptible to noise, and the authors of [1] highlighted the advantage of use of object-level fusion, which involves relatively less computational prowess and high immunity to noise. Thus, for real-time embedded systems, object-level sensor fusion techniques are more suited than low-level fusion. Work done by Badue et al. [22] highlights the benefit of using more than one sensor (which is also backed by Stampfle et al. [9]) and fusing their data at an object level. Accordingly, we implemented object-level multi-sensor fusion in the work described in this paper. These works were closely studied in order to understand the spatial synchronisation aspect of the LiDAR and camera sensor fusion.

Research done by De Silva et al. [23] sheds light on the factors involved in combining data from various sensors involving temporal and spatial synchronisation. In this work, a geometrical model is worked upon for spatial synchronisation of data. In our work, we use a similar model for converting a 3D point cloud bounding box into a 2D space and then fusing the camera and LiDAR sensor data together. In their work, the authors used a resolution-matching algorithm based on Gaussian process regression to estimate unreliable or missing data. To combat the problem of uncertain data, we used a tracker algorithm. Thus, we can say that our work is a further development of the framework used in the project undertaken by De Silva et al. [23]. Decisions that are undertaken in driverless cars need to be computed with the help of as many sensor inputs as possible. Moreover, these decisions must be made in the presence of uncertainties and noise that come with pre-processing algorithms and data acquisition methods. Work done by De Silva et al. [23] addresses these two issues surrounding automotive sensor fusion. Work done by Yang et al. [24] and Wan et al. [25] also describes the application of the unscented Kalman filter in target tracking for automotive-specific applications. Based on these works, we considered the use of the unscented Kalman filter (UKF) as a tracker method in our system.

Various classification schemes for sensor data fusion are discussed in the work done by Castanedo [8]. Several sensor fusion algorithms are classified based on different parameters such as type of data processed, type of output data, and structure of framework. Based on this work, we propose the two fusion algorithms that we shall be focussing on—centralised and decentralised fusion methods. The architectures of these two methods were primarily inspired from the work done by Castenado [8] and Grime et al. [26].

In their work, Stampfle et al. [9] describe the construction of a Robot Operating System (ROS)-based sensor fusion node. ROS is a meta-operating system and provides standard operating system services like contention management, hardware abstraction, and process management, alongside high-level functionalities like synchronous and asynchronous calls and centralised databases. Being language independent, it is possible to develop software modules in ROS in C++ as well as Python, which allows for freedom to use necessary software nodes off the shelf without converting the code into one standard language. ROS also allows for use of a 3D visualisation tool—RViz, which will be used extensively for this work to project the camera and LiDAR images (input as well as output). By studying the work done by Bernardin et al. [27], we critically analyse the performance of the sensor fusion algorithms used to drive the said EBA features. In this case, mean average precision (mAP) values are used to gauge the consistency of an algorithm. The false positives, false negatives, and true positives values required for the calculation of the mAP value are derived from the confusion matrix by comparing the output of the fusion algorithm against KITTI dataset’s ground truth data.

Sensor technology—sensors and sensor fusion methodologies—form a critical part of modern autonomous vehicles. As specified in the work done by Badue et al. [22], different sensors used in varied fusion architectures lead to substantially different performances. It is clear from the work done by Aeberhard et al. [28] that most original equipment manufacturers (OEMs) prefer the use of high-level data fusion architecture for implementing ADAS algorithms in vehicles. Aeberhard et al. [28] showcase this through experimental analysis performed on the BMW 5 Series vehicle. Accordingly, we also consider a high-level sensor fusion architecture for our work.

### 2.2. Classification of Sensor Fusion Methods

Sensor data fusion involves the consideration of fundamental parameters like the speed of operation of the fusion algorithm on an embedded platform, its accuracy, computational load, architecture, type of data at the input and output, type of sensor configuration, and, ultimately, the cost of implementation. Hence, it is imperative to thoroughly classify various sensor fusion techniques. By studying various works and projects, we broadly classify sensor fusion techniques according to some criteria, as shown in Table 1.

Object-level sensor fusion (by Luo et al. [34]) has significant advantages over raw data-based sensor fusion (low-level fusion) as it ensures modularity and allows for ease of benchmarking. Moreover, the fusion techniques can be relatively simple to develop. Studying sensor fusion architectures and differences in performance for multiple fusion methodologies is important since, when it comes to implementing sensor fusion algorithms on embedded systems [21], it is important to use an optimum architecture which gives acceptable accuracy.

Accordingly, in our work, we chose to use and analytically compare object-level ‘centralised’ and ‘decentralised’ fusion methods, as inspired from the work done by Castanedo [8] in order to drive the EBA features.

## 3. Proposed Fusion Methods

### 3.1. Fusion Architectures—Centralised and Decentralised Fusion

**Figure 1:**
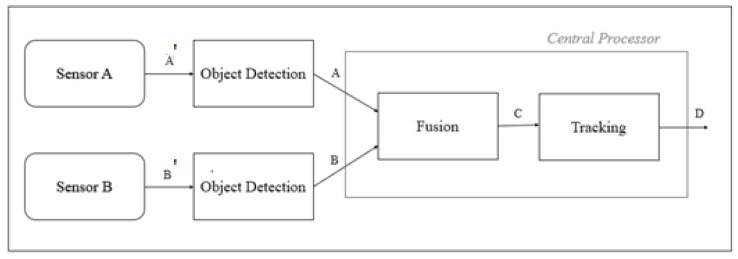
Architecture of OCSF.

In this paper, the centralised sensor fusion is referred as object-level centralised sensor fusion (OCSF) and the decentralised sensor fusion as (object-level decentralised sensor fusion (ODSF). The architecture of OCSF is shown in Figure 1.

The terminology used in Figure 1 is explained below:

A’, B’—Raw data from sensor (pixel-level data for camera and point cloud data for LiDAR)
A, B—Processed data from sensor object detection blocks. Pre-processing blocks indicate object detection algorithms implemented for the respective sensors.
C—Temporally and spatially synchronised data from the two sensors.
D—Fused data. Output of sensor fusion; these data are the output of the tracking algorithm, and are immune to false negatives, false positives, and other noise present in sensor data.

In OCSF, the fusion and tracking node is built inside the central processor. The fusion block receives synchronised data from various input blocks, which in this case are sensors A and B (camera and LiDAR, respectively). The output of the fusion block is given as the input to the tracker block. The tracker helps in suppressing noise, false positives, and false negatives, thereby providing fusion output with least errors.

**Figure 2:**
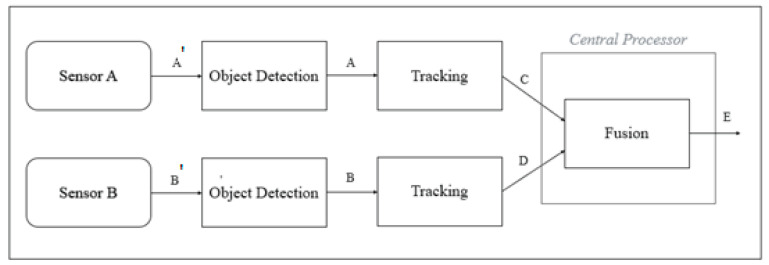
Architecture of ODSF.

The architecture of ODSF is shown in Figure 2.

The terminology used in Figure 2 is explained below:

A’, B’—Raw data from sensor (pixel-level data for camera and point cloud data for LiDAR).
A, B—Data from the sensor object detection blocks. Pre-processing blocks indicate object the detection algorithm implemented for the respective sensors.
C—Tracking data of Sensor A. This block ensures that data are consistent despite inconsistencies at the output of the pre-processing block.
D—Tracking data of Sensor B. This block ensures that data are consistent despite inconsistencies at the output of the pre-processing block.
E—Output of the fusion block. Data from both sensors are spatially and temporally aligned.

In ODSF, the fusion node is built inside the central processor; however, the tracking nodes for respective sensors are outside the central processor. The tracker, which is applied to both sensors, independently helps in suppressing false positives, noise, and false negatives for each sensor, thereby providing the central processor with data that are pure and devoid of errors and inconsistencies. As the tracker is applied independently to both sensors, it can be understood that this architecture involves higher processing and is computationally heavier than OCSF. However, as highly consistent data from both sensors are fed to the fusion algorithm, the output of the architecture is highly precise.

In both these methods, the pre-processing block comprises the respective object detection algorithms. The tracking block is the unscented Kalman filter used in both architectures (in a different manner, however). The alignment block takes care of the spatial and temporal alignment of data from the two sensors. The fusion block ultimately associates the data from the two sensors to a single fixed target.

The only difference between the two proposed methods is the way the ‘tracker’ block is used. As we shall later see in the experiments and results section, the position of the tracker block significantly affects the algorithm performance. In ODSF, the tracker is applied on individual sensor data before the data are fused, while in OCSF, the tracker is applied only once on the final fused output.

### 3.2. Components of the Proposed Fusion Methods

#### 3.2.1. Camera Object Detection

**Figure 3:**
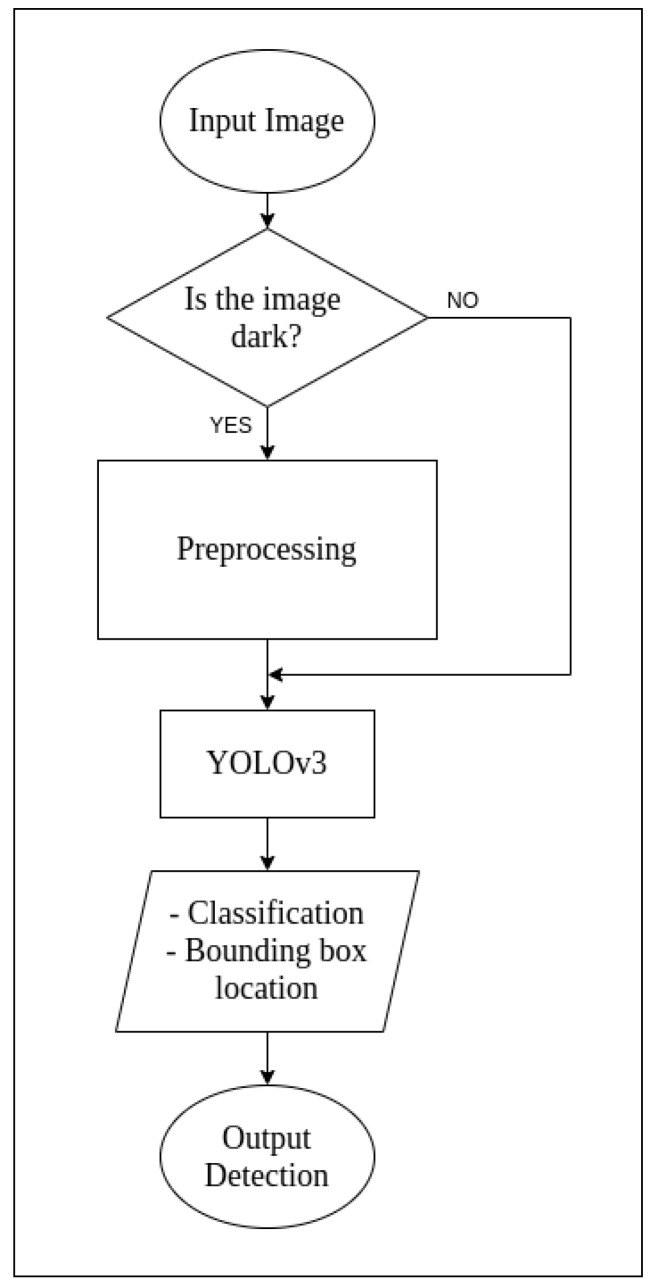
Flow diagram for YOLOv3.

You Only Look Once (YOLO) is a popular algorithm based on convolutional neural networks for detecting objects in a 2D image. It is not one of the most accurate algorithms, but it is very efficient in terms of accuracy and real-time detection [36,37]. Alongside predicting class labels, YOLO also detects the location of respective target objects within the image. For our application, we are not focusing on the class labels; however, this algorithm was chosen so that, if our work were to evolve in the future, such that object classes were to be made useful, no substantial changes would have to be made to the architecture. This algorithm divides the image into numerous smaller regions and predicts probabilities of object presence, and its bounding box within the selected region [37]. Figure 3 shows a high-level flow diagram for YOLOv3.

**Figure 4:**
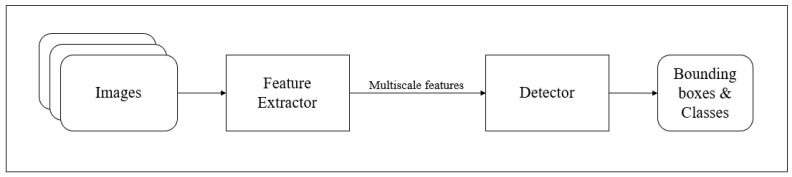
YOLOv3 network architecture.

Compared to the prior versions, YOLOv3 has a multi-scale detection and a much stronger feature extractor network, alongside changes in the loss function [38]. As a result, YOLOv3 has the capability to detect a multitude of targets, despite their size. Like any other single-shot detectors, this algorithm also makes real-time inference possible on standard CPU-GPU devices [37,38]. The network architecture for YOLov3 is as seen in Figure 4. In YOLOv3, a slightly tweaked architecture is used with the application of a feature extractor known as DarkNet-53. DarkNet-53 consists of 53 convolutional layers such that each layer is followed by Leaky ReLU activation and batch normalisation [38]. YOLOv3 is an open-source algorithm and was used off the shelf, as worked upon by Lee et al. [36].

#### 3.2.2. LiDAR Object Detection

**Figure 5:**
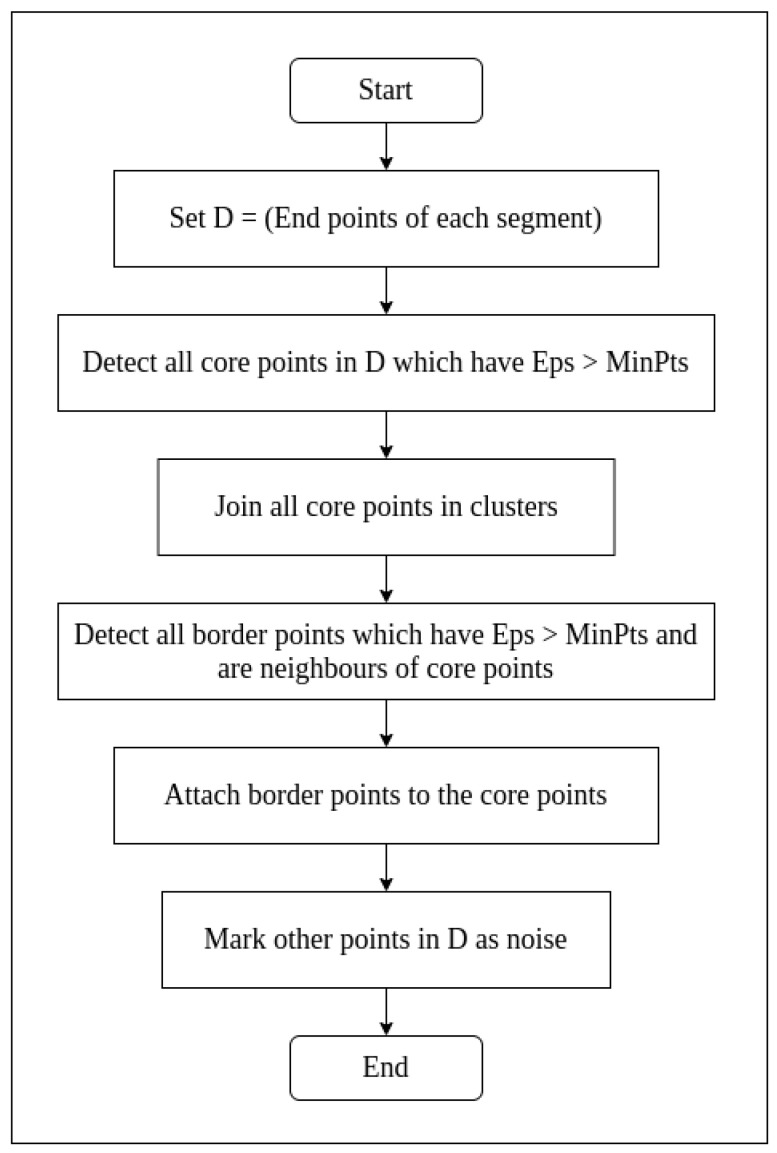
Flow chart of DBSCAN algorithm.

Density-Based Spatial Clustering of Applications with Noise (DBSCAN) is popular and, as the name suggests, a ‘density-based clustering non-parametric algorithm’ [39]. If a set of points are fed to its input, the algorithm works towards grouping points that are closely packed [40]. The algorithm marks such points as ‘inliers’. On the other hand, the points which lie outside the detected clusters are called ‘outliers’. In short, the DBSCAN algorithm separates high-density clusters from low-density point cloud pixels [41]. The flow chart for DBSCAN is as shown in Figure 5.

Here, ‘Eps’ is the maximum distance neighbourhood between points in a cluster, and ‘MinPts’ is the minimum size of points necessary to form a cluster.

The DBSCAN algorithm can be summarised in terms of input, output, and process as shown below:

**Input:** N objects to be clustered and some global parameters (Eps, MinPts)
**Output:** Clusters of objects
**Process:**

1. Select a point *p* arbitrarily.
2. Retrieve all density-reachable points from *p* with respect to Eps and MinPts.
3. If *p* is a core point, a cluster is formed.
4. If *p* is a border point, no points are density reachable from *p* and DBSCAN visits the next arbitrary point in the database.
5. Continue the process until all points in the database are visited.

**Figure 6:**
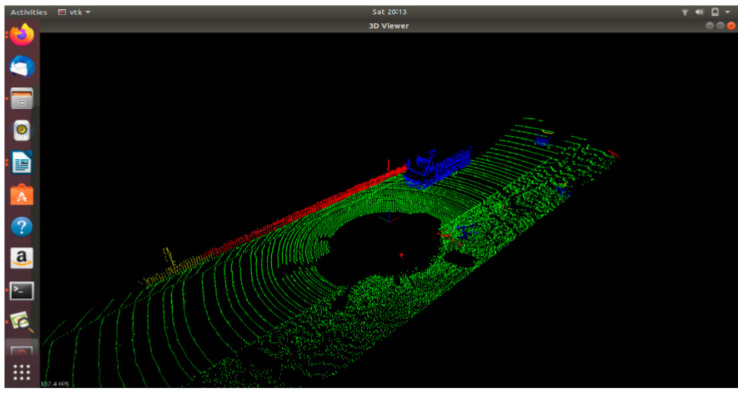
LiDAR point cloud: the points other than green represent high-density clusters.

**Figure 7:**
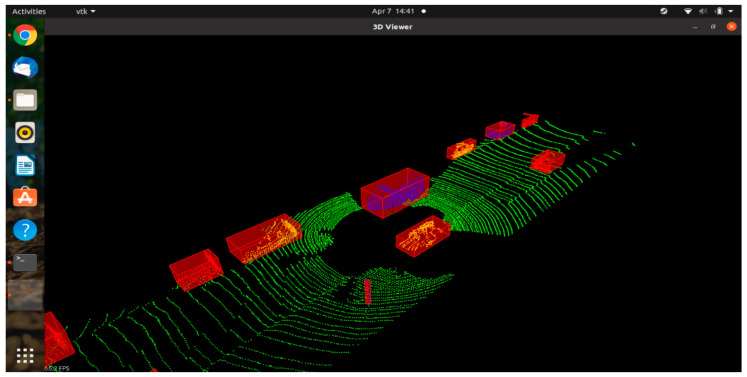
Bounding boxes drawn around the high-density clusters in LiDAR point cloud using DBSCAN.

In LiDAR point clouds, with a typical example that can be seen in Figure 6, the vehicles and objects on and beside the road are seen as high-density, closely packed clusters. The algorithm detects these clusters and draws a bounding box around them, as shown in Figure 7.

DBSCAN for LiDAR detection is an open-source algorithm and was used off the shelf, as worked upon by Ester et al. [42].

#### 3.2.3. Tracking

Tracking in automotive sensor fusion is the ability of the system to visualise and perceive various road objects around the vehicle and successfully track or follow them along the course of navigation. Detection and tracking are the core tasks in modern autonomous vehicles [24]. While detection algorithms help to create an object list of presence of various objects surrounding the ego-vehicle, tracking helps in understanding the way the obstacle or object has been moving and estimates the position of object in near future. Tracking algorithms are important because they help in combating the phenomenon of false positives and false negatives to a great extent [43]. As the past state of the object is always known, one can estimate the present state of the object even if the detection algorithm does not detect or falsely detects the present state of said object.

In our application, the tracker shall be exposed to highly non-linear inputs, as is in the case of realistic real-world cases. The detection algorithm may or may not always detect the obstacles (in case of false negatives) or can detect non-existent obstacles in few cases (in case of false positives). Furthermore, obstacles can occur and disappear outside of any control or pattern. When the system is nonlinear, the extended Kalman filter (EKF) tends to diverge [43], while unscented Kalman filter (UKF) tends to produce comparatively better results [24,43].

The unscented transformation is a method used to calculate the statistics and behaviour of any random variable subjected to nonlinear transformation. The unscented Kalman filter utilises a set of points to propagate them through the actual nonlinear function, instead of linearising the functions. The points to be fed to the filter are chosen such that their mean, covariance, and higher order moments match that of the Gaussian random variable. The mean and covariance can be recalculated using these propagated points to yield better and more accurate results compared to a Taylor Series function (which is fully linear). Here, sample points are not selected arbitrarily. In their work, Lee [44] demonstrated the superior performance gain of UKF over EKF for the estimation of state of the detected objects in highly non-linear systems. Thus, among the two considered predictors, we choose the UKF for our application.

**Figure 8:**
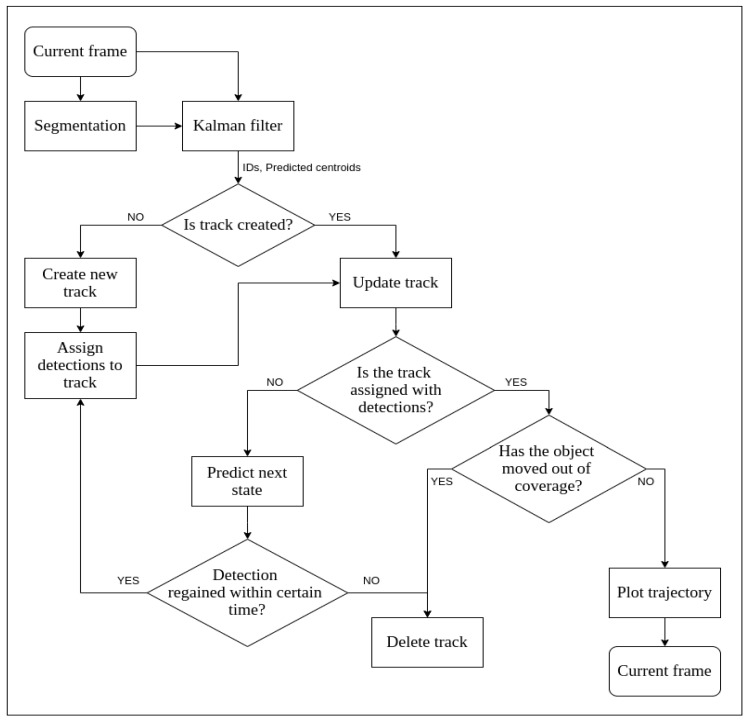
Flow chart for UKF implementation.

The flowchart for UKF implementation can be seen in Figure 8. The states that are implemented in UKF are as follows: (1) state predictor, (2) measurement predictor, and (3) state updater.

The UKF for position estimation of objects is an open-source algorithm and was used off the shelf as worked upon by Wan et al. [25].

#### 3.2.4. Data Fusion

##### Synchronicity of Data

This step applies to both OCSF and ODSF. In OCSF, data synchronicity is maintained before tracking, while in ODSF, data synchronisation is carried out after tracking blocks. An advantage of using the KITTI dataset (http://www.cvlibs.net/datasets/kitti/, (accessed on 31 July 2021) see Section 3.3 for details) is that the data are already temporally synchronised. As a result, we only take care of the spatial synchronisation of LiDAR and camera data.

To spatially synchronise the LiDAR and camera data (tracked and filtered in the case of ODSF, and unfiltered processed data in the case of OCSF), the calib_velo_to_cam.txt file provided in the KITTI dataset is used. This file consists of the rotation matrix and translation vector necessary to map the 3D LiDAR data onto the 2D image data. The spatial synchronisation part of the algorithm is done by the ‘alignment block’.

A 3D point ‘x’ in the 3D LiDAR space can be projected into a point ‘y’ into 2D camera space as shown in Equation (1) [43,45,46]:

Y = *p* × R × x (1)

where:

*p* is the Projection matrix after rectification:

(2) $$p=\left[\begin{array}{ccc} 721.53 & 0.00 & 609.55\\ 0.00 & 0.00 & 721.53\\ 172.85 & 0.00 & 0.00\\ 0.00 & 1.00 & 0.00 \end{array}\right]$$

where R is the rectifying rotation matrix. For rotation in a three-dimensional space, we can describe this as an anti-clockwise rotation by an angle θ about the *z*-axis. The 3 × 3 orthogonal matrix representing the transformation is given by:

(3) $$R=\left[\begin{array}{ccc} \cos\theta & -\sin\theta & 0\\ \sin\theta & \cos\theta & 0\\ 0 & 0 & 1 \end{array}\right]$$

**Figure 9:**
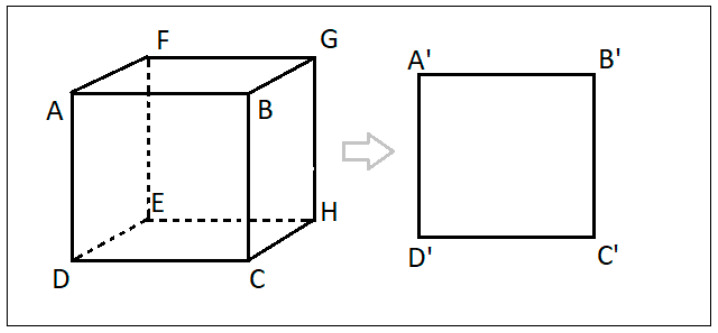
3D object bounding box as projected into corresponding 2D space.

Thus, by using Equation (1) with values from Equations (2) and (3) for *p* and R, respectively, we can associate the 3D LiDAR points with the 2D image points, as seen in Figure 9.

##### Executing Fusion Node—OSCF and ODSF

This step applies for both OCSF and ODSF. In OCSF, aligned and untracked noisy data are fed to the fusion node, while in ODSF, aligned tracked data are fed to the fusion node. However, the principles of operation and execution remain the same for both.

The objects detected by the camera object detection algorithm are identified by two parameters, namely:

1. Parameters of top left corner of the bounding box, that is, (x1, y1), and
2. Width and height of the bounding box, that is, (h, w).

**Figure 10:**
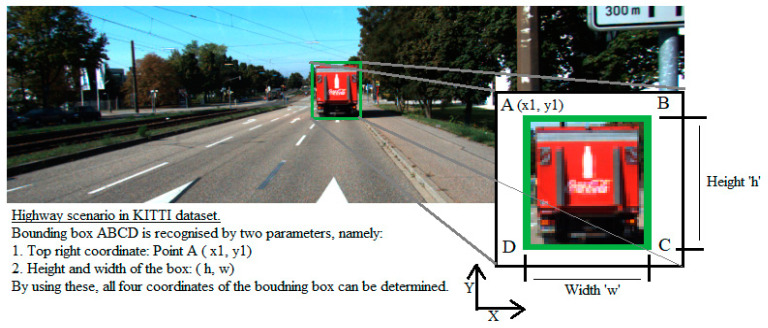
Description of a bounding box for a detected object in 2D space.

This can be understood from the details shown in Figure 10.

In Figure 10, consider the bounding box (ABCD). Accordingly, the cartesian coordinates of points A, B, C, and D can be as seen in Table 2.

Objects detected by the LiDAR object detection algorithm are also identified by two parameters, namely:

(1) Parameters of front top left corner of the bounding box, that is, (x1, y1, z1).
(2) Width, height, and depth of the bounding box, that is, (h, w, l).

The cartesian coordinates of points B, C, D, E, F, G, and H, as they can be derived, are shown in Table 3 (consider Figure 9 for the naming convention).

By using spatial transformation, every point in the LiDAR 3D space in Table 3 will be transformed into a respective point in the 2D camera space. Thus, after transforming 3D bounding boxes into the 2D space, we shall have a total of two 2D bounding boxes for each detected object—one bounding box is a result of camera object detection algorithm and the other one is the transformed output of the LiDAR object detection algorithm. If the transformation is accurate, and both sensors have detected the object with precision, the overlap between the two bounding boxes should be high. For this work, an intersection of union (IoU) value [11] of 0.7 was used, that is, the detection is considered as a true positive if more than 70% of the area of the 2D bounding boxes is overlapping.

**Figure 11:**
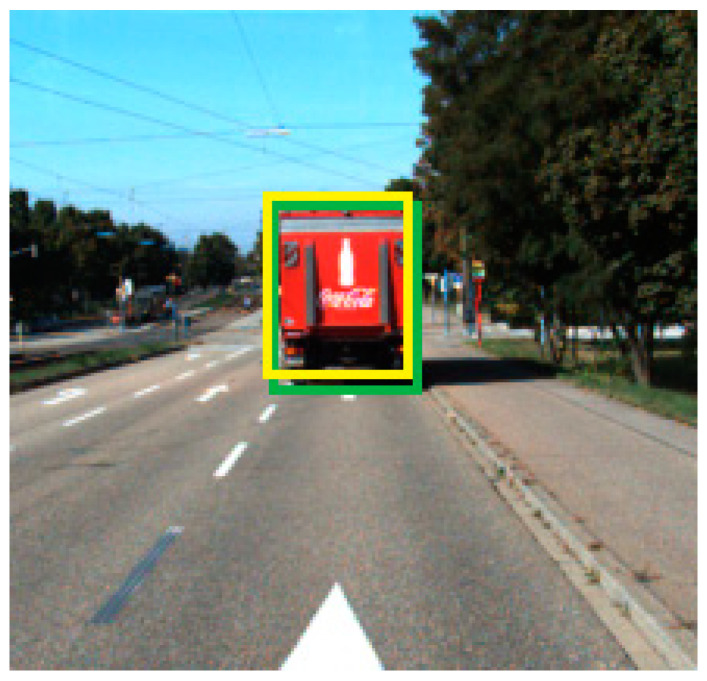
Camera (green bounding box) and transformed LiDAR bounding box (yellow) imposed in the 2D space.

These two bounding boxes can be seen in Figure 11. The yellow bounding box is the transformed LiDAR detection from 3D to 2D and the green bounding box is the camera-detected 2D box.

The fusion node associates camera data to the LiDAR data. The transformed bounding box detected by the LiDAR detection algorithm is associated on a pixel level with the bounding box detected by the camera detection algorithm. As the intersection over union (IoU) value is more than 0.7, the detections from the camera and LiDAR are fused together, and the transformed 2D bounding box detected by the LiDAR is considered as the final detection.

However, this technique works perfectly if both sensors provide reliable data with considerable accuracy. Bounding boxes of the two sensors can be associated only if both sensors detect an object. Data cannot be associated if one sensor picks an object and the other one fails to detect the same. For OCSF, where data are inconsistent at the input of the fusion node, consider a case as below:

1. Both sensors have detected an object, and the fusion node now associates their bounding boxes.
2. Some frames later, one of the two sensor detection algorithms gives a false negative detection and does not detect the object.

In this case, the fusion cannot be carried out and the fusion node provides a NULL output (which is similar to ‘No Object Detected’). This results in inconsistencies in the output of the fusion node. We then use the tracking node to tackle this problem for OCSF. In ODSF, however, as filtered data are received at the input of the fusion node, lesser anomalies are observed, and even if noise, false positives, or false negatives are present in the output of the camera and LiDAR object detection algorithms, the output of the fusion node is consistent, thanks to the tracking node, which is independently applied to both sensors before fusion. However, if inconsistent tracks are found in ODSF (different tracks for two different sensor outputs), the tracks are ignored, resulting in a NULL output. This is unexpected and would lead to an undesirable output from the fusion block.

### 3.3. Implementation of OSCF and ODSF

We have implemented the OCSF and ODSF architectures in the Robot Operating System (ROS)-based environment. For all implementation and experimentation, an Intel i5-based Ubuntu 18.04 machine was used. To make the system language agnostic, the Robot Operating System (ROS) is used. The KITTI dataset is used for the camera and LiDAR images used to develop and test the proposed algorithms. The advantage of using the KITTI dataset is the variety of testing data—for both camera and LiDAR—alongside being open source and having ease of compatibility for using the sensor data as they are [3]. The KITTI dataset also provides an easy method to convert the available to rosbag, thereby making it convenient to interface the data with a ROS environment.

#### Creating the ROS Environment

Using Robot Operating System (ROS) provides flexibility for development and helps in maintaining modularity. Multiple nodes can be added or removed without hassle and data can be easily debugged, envisioned, and processed. ROS provides cross-language development liberty and is language agnostic. As a result, the camera object detection algorithm in Python 3.6 and the LiDAR object detection algorithm developed in C++ can be integrated easily. In the Sensor_Fusion node, software nodes, as shown in Table 4, are used for both architectures (OCSF and ODSF). However, the order in which they are made to work differs.

An evaluation node is built for evaluating the performance of the fusion architectures. This node primarily gives an idea of the computational power required for implementing the architecture. Visualisation node is built to display the fused data on RViz, which is the visualisation tool used in ROS.

### 3.4. Examples for Sensor Data Fusion

**Figure 12:**
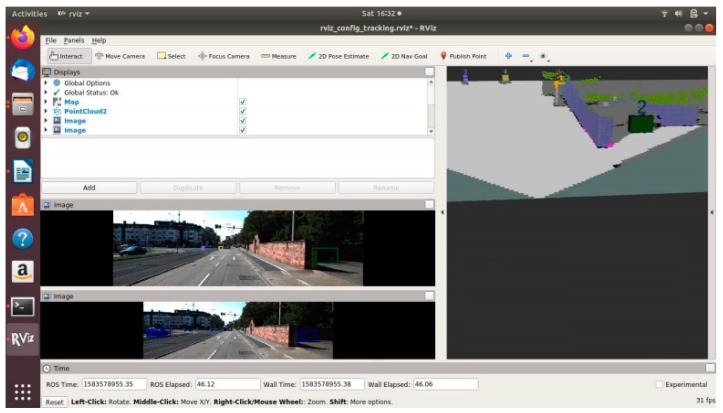
Output of sensor fusion in contrasting surroundings.

**Figure 13:**
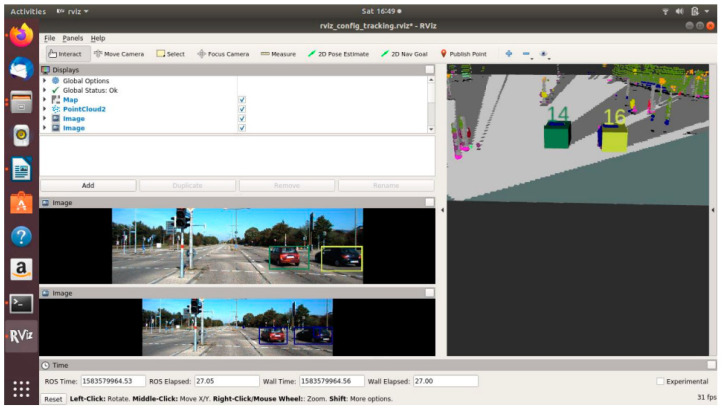
Output of sensor fusion in brightly lit surroundings.

**Figure 14:**
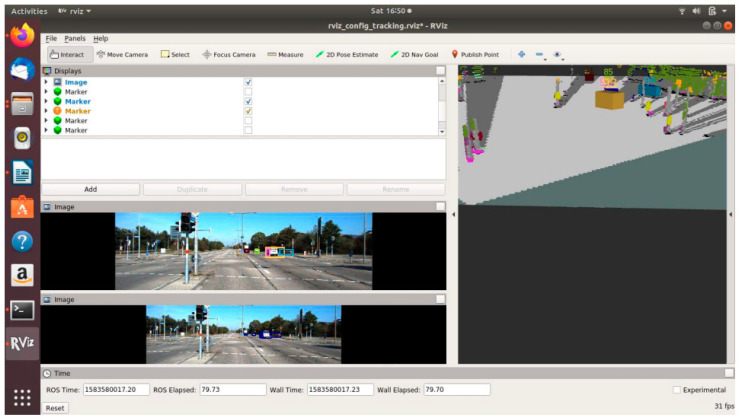
Output of sensor fusion in brightly lit surroundings when target objects are at a distance.

We tested the system in three scenarios for both OCSF and ODSF:

1. Detections in highly contrasting situations (bright roads and dark shadows)—Figure 12.
2. Detections in brightly lit scenarios—Figure 13.
3. Reliable detections at far distances in brightly lit scenarios—Figure 14.

Figure 12 shows that the fusion algorithm provides output as expected when exposed to highly contrasting scenes. Vehicles in darker parts of the image (the one with green bounding box) are detected well alongside the objects in the brighter parts of the image (with purple and yellow bounding boxes).

Figure 13 depicts the scenario in a bright sunny environment. Target objects in very bright surroundings are also detected properly.

Figure 14 depicts a scenario in which vehicles are far away from the ego vehicle. Such objects are also detected properly.

Thus, for both OCSF and ODSF, the qualitative performance of the algorithms seems acceptable under a myriad of circumstances. We shall now utilise these sensor fusion algorithms to gauge the performance of EBA.

## 4. Emergency Brake Assist (EBA) Using OCSF and ODSF

The output of central processor in the fusion framework is fed to the EBA application. The EBA is designed as worked upon by Ariyanto et al. [19], where ultrasonic sensors are used to detect any object in the vicinity of the vehicle. In this work, a similar feature is designed, except instead of ultrasonic sensors, fused data from camera and LiDAR are used to perceive the environment. The scenario shall be considered as an ‘Unsafe Scenario’ if the target object(s) detected is/are closer than 5 m in the driving path of the ego-vehicle.

**Figure 15:**
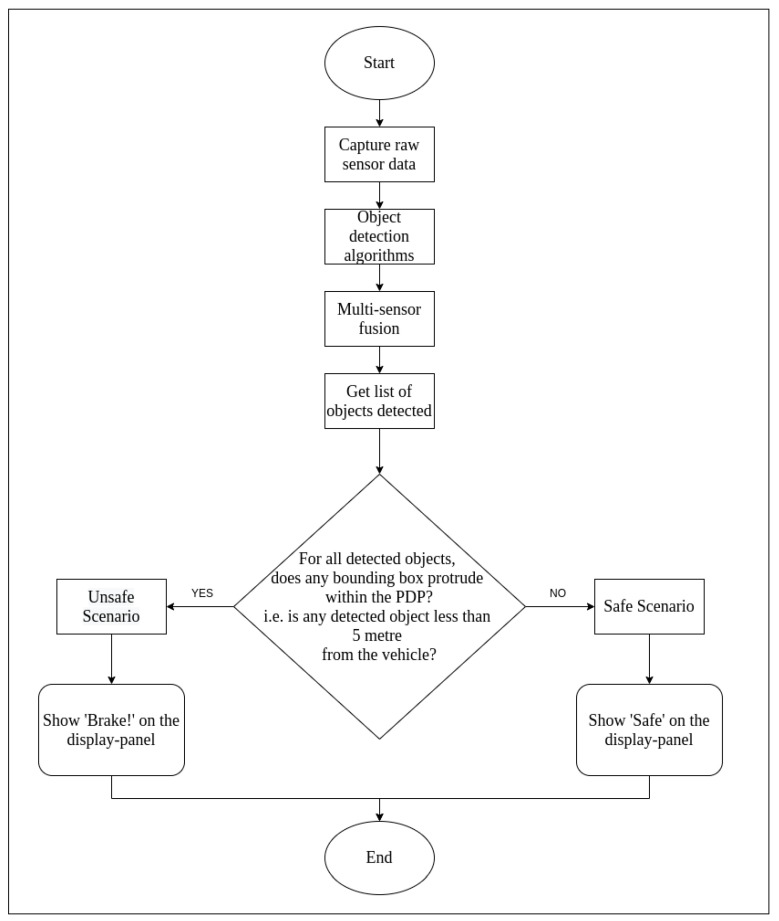
Flow chart for EBA functionality.

The projected driving path (PDP) is considered to be the area in front of the ego-vehicle with a width of 1.6 m (which is the width of the ego-vehicle) and length of 5 m. If any one of the four corners of the any detected bounding box of the target object(s) shall lie within the PDP, the EBA shall display ‘Brake!’ on the display window (thereby categorising the scenario as an ‘unsafe’ one). For safe scenarios, it shall display ‘Safe’ in the display window. The flow chart of functionality of this application is as seen in Figure 15.

### 4.1. Safe Scenario for EBA

Consider a detected bounding box whose four corners are P1 (x1, y1), P2 (x2, y2), P3 (x3, y3), and P4 (x4, y4).

**Figure 16:**
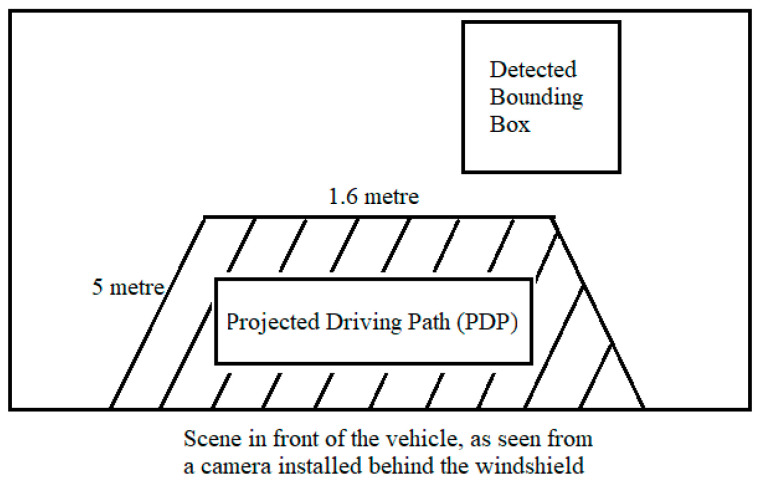
Safe scenario for EBA.

**Figure 17:**
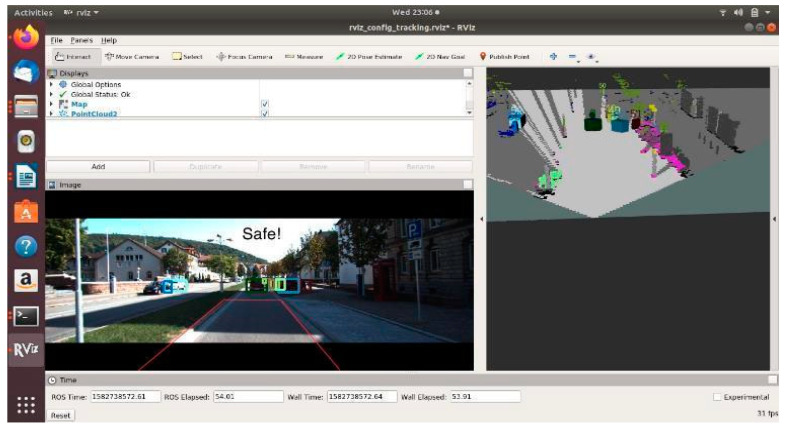
Safe scenario detected by OCSF.

As shown in Figure 16, the detected target bounding box does not protrude into the projected driving path (PDP). As the PDP is void of any target objects, the system considers it as a ‘safe’ scenario. Figure 17 shows a real-time representation of this scenario.

### 4.2. Unsafe Scenario for EBA

Consider a detected bounding box whose four corners are P1 (x1, y1), P2 (x2, y2), P3 (x3, y3), and P4 (x4, y4).

**Figure 18:**
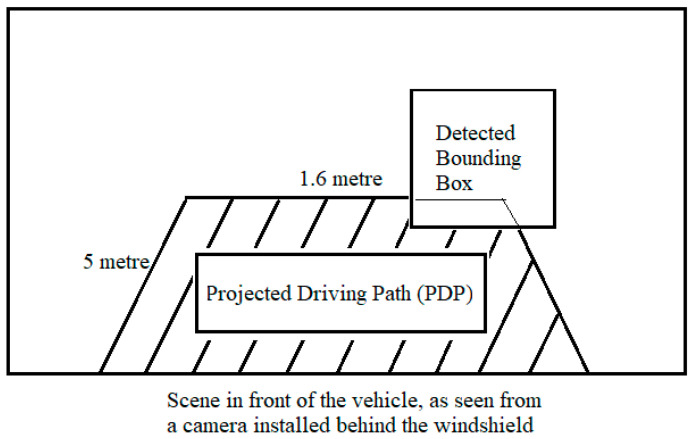
Unsafe scenario for EBA.

**Figure 19:**
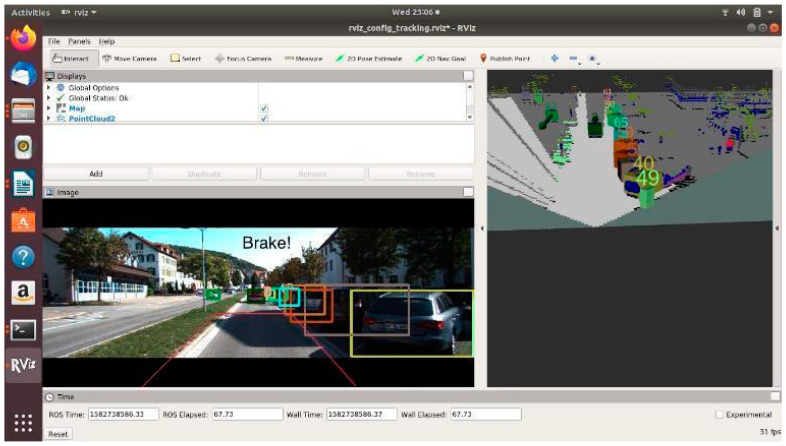
Unsafe scenario detected by OCSF.

As shown in Figure 18, the detected target bounding box does protrude into the PDP. As a result, the system considers it as an ‘unsafe’ scenario and displays ‘Unsafe!’ in the display window, as shown in Figure 19.

### 4.3. OCSF-Driven EBA

**Figure 20:**
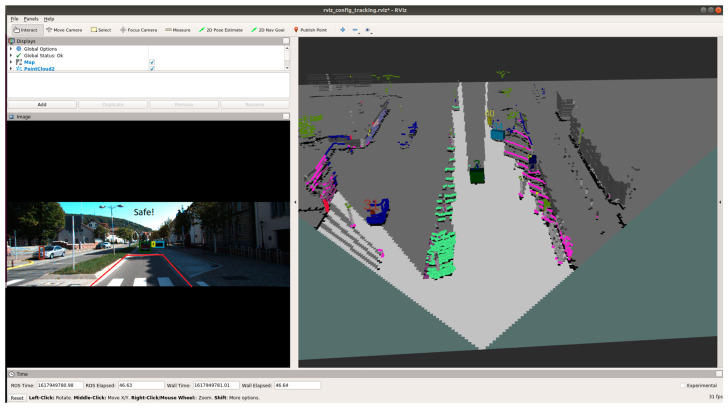
‘Safe’ scenario as detected by OCSF-driven EBA.

**Figure 21:**
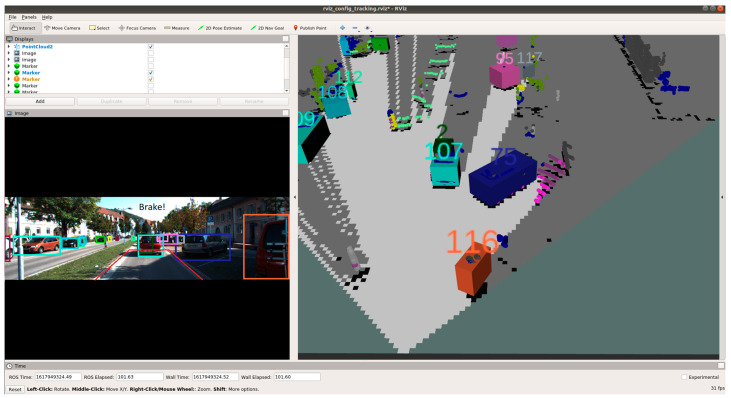
Unsafe scenario as detected by OCSF-driven EBA (object IDs are depicted above the 3D bounding boxes).

**Figure 22:**
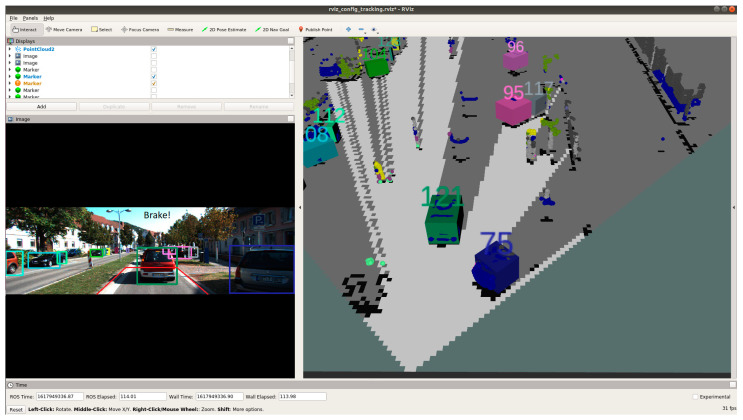
Unsafe scenario as detected by OCSF-driven EBA (object IDs are depicted above the 3D bounding boxes).

Figure 20, Figure 21 and Figure 22 show various safe and braking scenarios. In Figure 20, no objects are present in the PDP. As a result, the scenario is classified as a safe one.

In Figure 21 and Figure 22, road occupants are present inside the PDP. As a result, the scenario is classified as an unsafe one.

For all three circumstances shown in Figure 20, Figure 21 and Figure 22, the following observations are of particular interest.

1. The frame rate is consistent around 31 frames/s.
2. Instances of false positives or false negatives are observed at times, as expected from the OCSF-driven EBA algorithm.
3. The tracker algorithm does a good job of suppressing the false positives (FP) and false negatives (FN); however, not all FPs and FNs are filtered. It can be understood that the number of FPs and FNs would be considerably higher if the tracker were not used.

The PDP is a fixed area in front of the vehicle. As of now, the steering angle and vehicle speed do not affect the area under the PDP quadrilateral. However, as we are demonstrating the sensor fusion-based ADAS feature, this is an acceptable compromise, and this can be evolved in later versions of the work.

### 4.4. ODSF-Driven EBA

**Figure 23:**
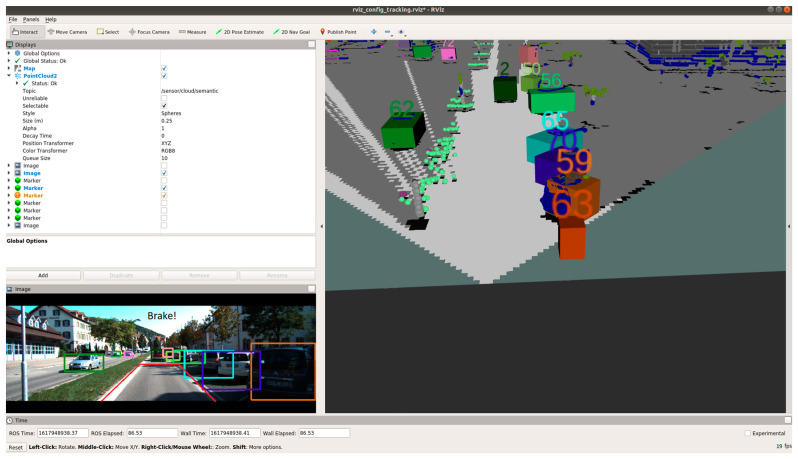
Unsafe scenario as detected by ODSF-driven EBA (object IDs are depicted above the 3D bounding boxes).

**Figure 24:**
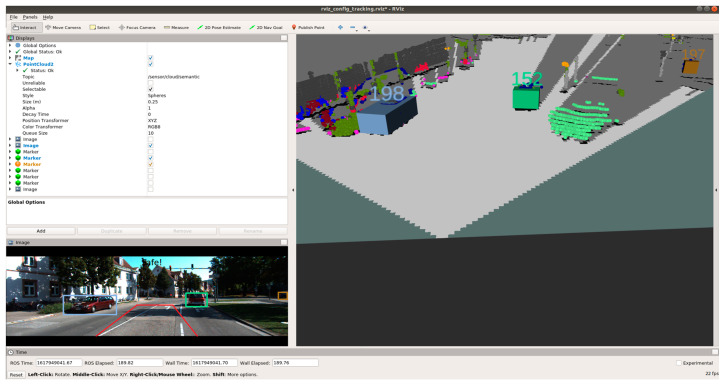
Safe scenario as detected by ODSF-driven EBA (object IDs are depicted above the 3D bounding boxes).

**Figure 25:**
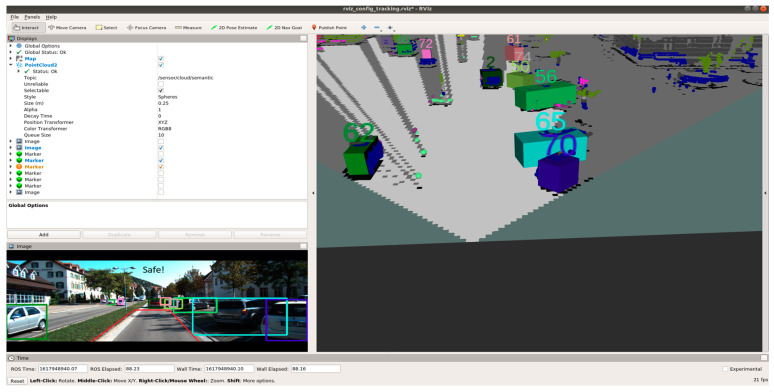
Safe scenario as detected by ODSF-driven EBA (object IDs are depicted above the 3D bounding boxes).

Figure 23, Figure 24 and Figure 25 show various safe and braking scenarios for ODSF-driven EBA. In Figure 23, one or more corners of the bounding boxes of vehicles parked alongside the road enter the PDP. As a result, the scenario is identified as an unsafe one.

Figure 24 and Figure 25 depicts a safe scenario in which no target objects are present in the PDP.

For all three circumstances shown in Figure 23, Figure 24 and Figure 25, the following observations are of particular interest.

1. The frame rate is consistent around 20 frames/s; thus, comparatively less frame rate is observed.
2. Compared to OCSF-driven EBA, lesser false positives and false negatives are observed, as expected from the ODSF-driven EBA.
3. In this case, the tracker algorithm suppresses the false positives and negatives. As these FPs and FNs are suppressed at a modular level before the data are fused, the accuracy of this method is much higher than the OCSF-driven EBA.
4. Like in the previous method as well, the steering angle and vehicle speed do not affect the area under the PDP quadrilateral. However, as we are demonstrating the sensor fusion-based ADAS feature, this is an acceptable compromise, and this can be evolved in later versions of the work.

### 4.5. Results

Object-level centralised and decentralised sensor fusion can thus be successfully used to drive the said ADAS algorithm. Furthermore, through experiments, we also conclude that both fusion techniques provide a higher qualitative performance compared to mono-sensor systems. For benchmarking of the mono-sensor system, we consider the EBA-driven by camera sensor alone. While there are some imminent drawbacks with camera-driven EBA, such as lesser reliability of the system in low-light conditions, we consider a scenario under perfect lighting for the sake of comparison. For experimental analysis, more than 100,000 frames of the KITTI dataset in urban, semiurban, and highway scenarios were considered. Various objects, such as commercial, heavy, and light on-road vehicles, pedestrians, and other relevant road objects were considered.

#### 4.5.1. Frame Rate for the Execution of EBA

The execution speed of an algorithm is a direct indication of the computational load that is incurred by the software on the system on which it is executed. Both fusion algorithms provide acceptable speed (~20 fps for ODSF and ~30 fps for OCSF). For the mono-sensor system, the frame rate is highest at ~37 fps. For the execution of multiple videos under different circumstances, Table 5 shows the frame rates observed for EBA driven by both fusion methods and mono-sensor architecture (the videos are chosen from the KITTI dataset).

The frame rate of EBA executed with OCSF is ~50% higher than EBA executed with ODSF, while the frame rate of EBA executed using mono-sensor architecture is ~35% higher than the one executed using OCSF. The prime reason for higher execution speed of OSCF- as compared to ODSF-driven EBA is because the computationally heavy tracker algorithm is implemented only once in OCSF, whereas in ODSF, it is implemented twice (once for each sensor output) for a single frame. In mono sensor-driven EBA, a higher frame rate is observed as a processing of only one sensor has to be done. The time profiling for EBA executed with both sensor fusion methods is as given in Table 6 and Table 7, respectively.

On the current system, where the CPU does not allow for much parallelisation of tasks, a stark difference between the performance of two architectures can be seen. However, if a capable embedded platform like NVIDIA Drive AGX [47], which has numerous GPU cores to allow for parallelisation of independent tasks, the ODSF can be implemented as fast or nearly as fast as the OCSF [30,48].

It can be seen from Table 6 and Table 7 that the tracker algorithm is computationally heavier than all other components in the system. The UKF algorithm consists of many approximations and iterations, because of which it is expected to be computationally heavy [49,50,51,52,53]. Other alternatives, such as the extended Kalman filter, might be computationally lighter but are prone to more errors [54].

#### 4.5.2. Accuracy and Precision of EBA

The tracklets.xml file in the KITTI dataset contains ground truth data for all instances. For both fusion methods, OCSF and ODSF, we store the bounding box data in the /evaluation folder. Later, the contents in the /evaluation folder are compared with data in tracklets.xml to get an idea of the accuracy of each architecture. A Python script is written to compare the objects detected by the fusion algorithm against the ground truth data. The IoU measures the overlap of the two bounding boxes under consideration—the ground truth box and the actual detected bounding box. For the current project, an IoU of 0.7 was considered in calculating the accuracy and precision of the detection fusion algorithms. A detected bounding box (the output of the fusion algorithm) is considered as a true positive if the IoU with the ground truth data are greater than 0.7. By calculating the true positives, false positives, and false negatives values, the mAP values for the OCSF output, the ODSF output, and the mono-sensor output for IoU of 0.7 for four separate videos were obtained and are listed in Table 8. If the IoU threshold is increased, the mAP values decrease accordingly for both OCSF and ODSF; however, the IoU threshold is set at 0.7 for optimum results.

As elaborated in Section 2.1, the false positives (FP), false negatives (FN), and true positives (TP) values are calculated by comparing the output of the fusion algorithm against the KITTI dataset’s ground truth data. mAP values are then calculated using the TP, FP, and FN values. The prime downside of the mono-sensor system can be observed from Table 8. For the mono-sensor architecture, the mAP value in all tested scenarios is less than half of the mAP value for the fusion architectures. Thus, despite the high frame rate, as seen in Table 5, the application of the mono-sensor architecture is not preferred due to extremely poor accuracy. The higher value of accuracy for ODSF is justified in ODSF, as noise (false positives, false negatives, and ghost object detections, etc.) is suppressed earlier than OCSF by using tracker immediately after detection algorithm. As a result, the data fed to sensor fusion node are already filtered and the effects of noise are nullified beforehand. Thus, the fusion algorithm can operate with minimal error, thereby providing more accurate results. Even if it is computationally heavier, ODSF provides more accurate results.

In general, the errors in the output of the fusion block are observed when either sensor fails to detect the object. This is the major reason behind lower performance of the OCSF. This inconsistency in the detection or false positive detections can be referred to as ‘Noise’. For ODSF, however, when an object is not detected, or falsely detected for few frames by any sensor, the respective tracker predicts its right position, and it drives the algorithm accordingly for the next frames.

Hence, if the ADAS algorithm needs to be run in situations where accuracy is of utmost importance, and cost of hardware platform is a second priority, ODSF shall provide for a better solution. It can thus be understood that OCSF is less immune to sensor noise and errors. As a result, at several instances, false negatives and false positives are observed in the sensor fusion output for OCSF. This error directly corresponds to a failure of the EBA under critical situations.

#### 4.5.3. Computational Cost of EBA

OCSF can be implemented on hardware platforms with fewer resources/lesser computational prowess, while ODSF requires platforms with more resources/high computational prowess to achieve the real-time performance. As ODSF is computationally heavier than OCSF, the cost of execution of OCSF is lesser than that of ODSF if a real-time application, such as EBA, is to be implemented using these methods. When executed on a computer with NVIDIA GeForce 1080 GTX Graphics card, OCSF was seen to be running at 46 fps and ODSF at 42 fps. Thus, on higher end machines, the performance of the two algorithms is on par with each other.

The computational cost of implementation of ODSF increases proportionally with the number of sensors in the system. However, the ability of the system to tackle more noise brought in by more sensors is also strengthened. Thus, depending on the criticality, budget, and nature of application of the target ADAS system, this can either be an advantage or disadvantage of ODSF compared to OCSF. Thus, on a general hardware platform with limited resources, EBA executed using OCSF shall still provide acceptable output, while EBA using ODSF shall show degraded performance (in terms of frame rate and hardware resources consumed). To obtain a real-time performance from EBA using ODSF, more expensive hardware will be required, while the same is not necessary for EBA using OCSF.

## 5. Conclusions

While it can be seen from Table 5 that mono-sensor-driven EBA provides very high frame rate, Table 8 proves that this high execution speed comes at the price of very poor accuracy. In ADAS applications, we need to attain a balance between the speed of execution and the accuracy of the system. Thus, due to substantially degraded accuracy of the mono-sensor system, fusion-based systems are preferred, thanks to their higher accuracy and acceptable execution speed. Even the least accurate version of sensor fusion-driven EBA (from the two methods stated in this paper) is more reliable and worthy than EBA driven by a mono-sensor system. Fusing data from multiple sensors might add to the cost of the system; however, the accuracy, precision, and reliability of the ADAS algorithm increase manifold, which, in turn, justify the higher cost of the fusion algorithm.

Considering the accuracy, computational load, and cost of execution of the two sensor fusion methods for driving EBA, we can say that both OCSF and ODSF have their respective advantages and disadvantages. While OCSF is simpler to execute and is computationally lighter, it provides comparatively less accuracy (as seen in Table 8); ODSF is more accurate than OCSF and has a better immunity to noise; however, it is computationally heavy and, hence, has a higher computational cost. Mono-sensor systems, on the other hand, are very light computationally; however, they also provide very poor accuracy. If an ADAS algorithm needs to be run on a less-expensive embedded platform, which has lesser hardware resources (a smaller number of CPU and GPU cores and less cache and RAM), like a Cortex-M-based STM32 platform [55,56], and less accuracy of EBA is acceptable, OCSF shall prove to be a comparatively better option. However, if hardware resources and computational cost are not a concern, and the accuracy and precision of the ADAS algorithm utilising the fusion architecture is of the utmost importance, ODSF is a more favourable option for driving EBA [57,58,59,60].

In real-world on-vehicle scenarios, if EBA is executed in L1–L2 automated vehicles [61,62], where the driver shall be expected to always remain attentive and in control of the vehicle, EBA driven by OCSF might be a beneficial option; however, if the vehicle is automated to L3 or higher, where the driver is not always expected to be attentive or in control of the vehicle, EBA driven by ODSF shall certainly be a more reliable and better alternative [63].

## Figures and Tables

**Table 1:** Classification of sensor fusion techniques.

Sr. No.	Criteria	Reference
1	Classification based on relation between the different input sources, which can be: Cooperative Complimentary Redundant	Whyte et al. [29] Chavez-Garcia et al. [30] Steinhage et al. [31]
2	Classification based on data types of input and output data, which can be: Data In-Data Out Data In-Feature Out Feature In-Feature Out Feature In-Decision Out Decision In-Decision Out	Dasarathy et al. [32] Steinhage et al. [31] Heading et al. [33]
3	Classification based on abstraction level of fused data, which can be: Raw Data (Low level) Decision or Object (High level)	Luo et al. [34] Chavez-Garcia et al. [30]
4	Classification based on type of fusion architecture: Hierarchical Distributed Clustered	Castanedo [8] Makarau et al. [35] Heading et al. [33]

**Table 2:** Cartesian coordinates for the 2D bounding box with top left corner A (x,y), height h, and width w.

Sr. No.	Point	Coordinates
1	A	(x1, y1)
2	B	(x1 + w, y1)
3	C	(x1 + w, y1—h)
4	D	(x1, y1—h)

**Table 3:** Cartesian coordinates of the 3D bounding box.

Sr. No.	Point	Coordinates
1	A	(x1, y1, z1)
2	B	(x1 + w, y1, z1)
3	C	(x1 + w, y1—h, z1)
4	D	(x1, y1—h, z1)
5	E	(x1, y1—h, z1 + l)
6	F	(x1, y1, z1 + l)
7	G	(x1 + w, y1, z1 + l)
8	H	(x1 + w, y1—h, z1 + l)

**Table 4:** Description of ROS nodes for OCSF and ODSF.

Sr. No.	ROS Node	Description
1	Detection_LiDAR	This node performs object detection on LiDAR data
2	Detetcion_Camera	This node performs object detection on camera data
3	Sensor_Sync	This node applies transformation matrix and statially synchronises LiDAR and camera data
4	Sensor_Fusion	This node associates the synchronised LiDAR and camera data together, thereby creating an object list which includes data from both the camera and LiDAR
5	Tracking	This node performs functionality of the unscented Kalman filter. The UKF is implemented on fused data for OCSF and independently on sensor data in ODSF.

**Table 5:** Frame rate for multiple videos for OCSF- and ODSF-driven EBA.

Experiment Number	Scenario	Frame Rate for EBA with OCSF	Frame Rate for EBA with ODSF	Frame Rate for EBA with Mono-Sensor
1	Densely populated urban street	32 fps	18 fps	36 fps
2		32 fps	20 fps	37 fps
3	Moderately populated urban	33 fps	20 fps	37 fps
4		31 fps	20 fps	37 fps
5	Sparsely populated highway	32 fps	22 fps	39 fps
6		32 fps	21 fps	38 fps
7	Densely populated highway	32 fps	21 fps	37 fps
8		32 fps	20 fps	38 fps

**Table 6:** Time profiling for EBA driven with OCSF.

Sr. No.	Software Block—OCSF	Time Taken for Execution (ms)
1	LiDAR object detection—DBSCAN	4
2	Camera object detection—YOLOv3	5
3	Alignment—Temporal and spatial data synchronisation	3
4	Data Fusion—Association of target objects	2.5
5	Tracking—UKF	16
6	EBA	2
**TOTAL**		**32.5**

**Table 7:** Time profiling for EBA driven with ODSF.

Sr. No.	Software Block—ODSF	Time Taken for Execution (ms)
1	LiDAR object detection—DBSCAN	4
2	Camera object detection—YOLOv3	5
3	Tracking for LiDAR detection—UKF	16.2
4	Tracking for camera detection—UKF	16.5
5	Alignment—Temporal and spatial data synchronisation	3
6	Data Fusion—Association of target objects	1.8
7	EBA	2
**TOTAL**		**48.5**

**Table 8:** mAP values for different videos for OCSF, ODSF, and mono-sensor output.

Experiment Number	Scenario	OCSF mAP (%)	ODSF mAP (%)	Mono-Sensor mAP (%)
1	Densely populated urban street	57.7857	63.9002	30.323
2		54.3361	64.7871	29.8019
3	Moderately populated urban	57.0128	66.6676	31.7009
4		58.9919	65.0118	33.2245
5	Sparsely populated highway	64.0089	70.5824	32.2637
6		64.3327	69.9066	33.034
7	Densely populated highway	62.7008	68.6842	30.8026
8		61.1029	67.7183	29.0807

## Data Availability

Not applicable.
